# Supplementary material for: Do workers accumulate resources during continuous employment and lose them during unemployment, and what does that mean for their subjective well-being?
Source: PLoS One. 2021 Dec 23;16(12):e0261794. doi: 10.1371/journal.pone.0261794 (PMC8699683; doi:10.1371/journal.pone.0261794)
Supplement: S1 Table — (PDF) [file pone.0261794.s001.pdf]

Between-level variables and correlations	1	2	3	4	5	6	7	8
1. Life satisfaction								
2. Emotional well-being (latent)	0,576							
3. Income (logged)	0,282	0,102						
4. Financial worries	-0,545	-0,408	-0,388					
5. Perceived employability	0,199	0,045	0,073	-0,164				
6. Frequency of socializing	0,279	0,063	0,113	-0,149	0,344			
7. Loneliness	-0,481	-0,662	-0,095	0,284	-0,045	-0,112		
8. Social support availability	0,226	0,221	0,033	-0,138	-0,002	0,085	-0,398	
9. Mastery (latent)	0,732	0,507	0,283	-0,590	0,482	0,411	-0,428	0,074
10. Unemployed at all occasions	-0,034	0,008	-0,072	0,048	-0,011	-0,016	0,006	-0,009
11. Number of employment spells	-0,100	-0,050	0,032	0,075	-0,017	-0,096	-0,024	0,006
12. Number of unemployment spells	-0,175	-0,052	-0,169	0,195	-0,050	-0,109	0,048	-0,015
13. Average employment duration	-0,007	0,105	0,143	-0,074	-0,362	-0,306	-0,153	0,064
14. Average organizational tenure	0,025	0,091	0,163	-0,116	-0,390	-0,253	-0,157	0,079
15. Average unemployment duration	-0,112	-0,024	-0,140	0,130	-0,069	-0,091	0,039	-0,023
16. Age	-0,056	0,081	0,128	-0,080	-0,553	-0,500	-0,064	0,063
17. $Age^2/10$	0,131	0,032	-0,072	-0,094	-0,001	0,301	0,102	-0,026
18. East German	-0,226	-0,046	-0,165	0,263	-0,133	-0,148	0,028	0,041
19. Female	0,029	-0,301	0,016	0,038	0,028	0,047	0,130	0,035
20. Average educational attainment	0,095	0,040	0,420	-0,227	0,064	0,023	-0,077	0,057
21. Rate of part-time employment	0,064	-0,061	0,061	-0,049	0,014	0,003	0,001	-0,008
22. Rate of marginal employment	0,089	-0,047	0,009	-0,100	0,011	0,094	0,116	-0,004
23. Rate of fixed-term employment	0,015	-0,047	-0,041	0,062	0,160	0,199	0,085	-0,083
24. Average overtime hours	-0,015	0,039	0,182	-0,030	0,089	-0,053	-0,069	-0,022
25. Rate of self-employment	0,014	0,041	0,098	-0,040	-0,028	-0,068	-0,056	0,015
26. Average occupational autonomy	0,111	0,091	0,439	-0,251	-0,059	-0,120	-0,185	0,085
27. Average number of prior unemployment spells	-0,163	-0,061	-0,126	0,171	-0,031	-0,100	0,087	-0,062
28. Average duration of prior unemployment	-0,149	-0,043	-0,148	0,171	-0,101	-0,132	0,091	-0,065
29. Average satisfaction with health	0,635	0,468	0,111	-0,277	0,286	0,282	-0,361	0,072
30. Disability at all occasions	-0,096	-0,027	-0,035	0,008	-0,149	-0,094	0,111	0,032

9	10	11	12	13	14	15	16	17	18	19	20
-0,056											
-0,075	-0,104										
-0,162	0,136	0,384									
-0,175	-0,070	0,163	-0,094								
-0,158	-0,065	0,185	-0,097	0,801							
-0,142	0,401	0,079	0,447	-0,086	-0,083						
-0,358	-0,013	0,124	0,021	0,586	0,550	0,037					
0,165	-0,011	-0,259	-0,120	-0,364	-0,278	-0,079	-0,292				
-0,097	-0,005	0,095	0,150	0,000	-0,035	0,073	-0,017	-0,005			
-0,016	-0,005	-0,032	0,006	-0,202	-0,159	0,013	-0,042	0,002	0,003		
0,195	-0,043	0,088	-0,061	0,066	0,082	-0,062	0,098	-0,213	0,049	-0,031	
-0,027	-0,041	0,054	-0,045	-0,035	0,000	-0,038	0,103	-0,109	-0,128	0,308	-0,011
0,040	-0,056	-0,069	-0,079	-0,292	-0,237	-0,065	0,019	0,379	-0,077	0,127	-0,041
0,140	-0,051	0,014	0,002	-0,258	-0,257	-0,032	-0,353	0,290	0,059	0,020	-0,052
0,100	-0,053	0,097	-0,063	0,187	0,118	-0,069	0,046	-0,229	-0,005	-0,213	0,213
0,044	-0,027	0,062	-0,046	0,134	0,107	-0,038	0,164	-0,077	-0,043	-0,079	0,118
0,135	-0,144	0,224	-0,200	0,451	0,439	-0,189	0,337	-0,459	-0,065	-0,117	0,553
-0,132	0,042	0,272	0,291	-0,114	-0,134	0,145	0,043	-0,147	0,097	-0,010	-0,034
-0,176	0,042	0,132	0,330	-0,117	-0,110	0,205	0,133	-0,114	0,084	0,021	-0,072
0,552	-0,004	-0,046	-0,076	-0,124	-0,104	-0,063	-0,292	0,105	-0,059	-0,014	0,070
-0,122	-0,002	-0,171	-0,058	-0,059	-0,050	-0,025	0,122	0,075	-0,028	-0,041	-0,057

**21**	**22**	**23**	**24**	**25**	**26**	**27**	**28**	**29**	**30**
-0,089									
-0,022	-0,048								
-0,121	-0,227	-0,037							
-0,009	-0,019	-0,118	0,066						
0,030	-0,220	-0,282	0,371	0,293					
-0,016	-0,037	0,049	0,012	-0,018	-0,074				
-0,016	0,071	0,012	-0,057	-0,019	-0,130	0,475			
-0,013	-0,032	0,096	0,025	-0,010	0,026	-0,088	-0,108		
-0,002	0,100	-0,029	-0,079	-0,033	-0,079	-0,002	0,034	-0,283	

Within-level variables and correlations	1	2	3	4	5	6	7	8	9
1. Life satisfaction									
2. Emotional well-being (latent)	0,525								
3. Income (logged)	0,073	0,035							
4. Financial worries	-0,231	-0,265	-0,123						
5. Perceived employability	0,101	0,094	-0,058	-0,152					
6. Frequency of socializing	0,052	0,069	-0,040	-0,012	0,042				
7. Loneliness	-0,197	-0,371	0,032	0,112	-0,068	-0,051			
8. Social support availability	0,131	0,140	-0,095	-0,063	0,071	0,075	-0,070		
9. Mastery (latent)	0,452	0,515	0,019	-0,432	0,233	0,070	-0,279	0,019	
10. Unemployment occasion	-0,153	-0,120	-0,171	0,144	-0,092	0,013	0,039	-0,036	-0,136
11. First 6 months in current job	0,002	0,004	-0,057	0,009	0,028	0,017	-0,013	0,015	0,035
12. Last year in current job	-0,031	-0,042	-0,028	0,029	0,013	0,021	-0,006	0,027	-0,022
13. First 6 months of current unemployment	-0,089	-0,073	-0,105	0,082	-0,033	0,008	0,013	-0,001	-0,069
14. Last year of current unemployment	-0,097	-0,078	-0,110	0,090	-0,071	0,011	0,025	-0,030	-0,090
15. 2nd employment spell	-0,007	0,009	0,063	-0,021	-0,030	-0,027	0,021	-0,043	0,003
16. 3rd employment spell	-0,009	0,002	0,040	-0,001	-0,041	-0,026	0,026	-0,054	-0,015
17. 4th and later employment spells	-0,014	-0,008	0,029	-0,001	-0,037	-0,021	0,021	-0,031	-0,017
18. 2nd unemployment spell	-0,081	-0,067	-0,082	0,078	-0,048	0,007	0,026	-0,040	-0,081
19. 3rd unemployment spell	-0,051	-0,041	-0,049	0,056	-0,031	0,001	0,019	-0,032	-0,052
20. 4th and later unemployment spells	-0,041	-0,032	-0,041	0,041	-0,038	0,002	0,009	-0,015	-0,060
21. Employment duration	-0,028	-0,032	0,278	-0,019	-0,146	-0,050	0,052	-0,120	-0,103
22. Organizational tenure	-0,049	-0,049	0,269	-0,006	-0,183	-0,044	0,061	-0,145	-0,127
23. Unemployment duration	-0,008	-0,004	-0,006	0,013	-0,018	-0,002	0,010	-0,029	-0,012
24. Age	-0,086	-0,082	0,410	0,003	-0,239	-0,098	0,122	-0,267	-0,195
25. $Age^2/10$	0,025	0,001	-0,115	-0,027	-0,048	0,071	-0,011	0,064	0,025
26. Educational attainment	-0,013	0,002	0,140	-0,011	0,009	-0,030	0,015	-0,051	-0,010
27. Part-time employment	-0,005	0,005	-0,051	-0,006	0,004	0,001	0,004	0,031	-0,012
28. Marginal employment	-0,043	-0,027	-0,122	0,050	-0,016	0,028	0,021	-0,008	-0,036
29. Fixed-term contract	0,002	0,001	-0,051	0,022	-0,003	0,009	0,003	0,008	0,006
30. Overtime hours	0,019	0,005	0,068	-0,031	0,030	-0,020	0,005	-0,009	0,036
31. Self-employment	-0,005	-0,007	0,040	0,029	-0,019	-0,028	-0,011	-0,036	0,031
32. Occupational autonomy	0,062	0,049	0,210	-0,078	0,016	-0,024	-0,015	-0,038	0,064
33. Number of prior unemployment spells	-0,017	-0,025	0,163	0,005	-0,059	-0,056	0,055	-0,140	-0,059
34. Total duration of prior unemployment	-0,014	-0,022	0,068	0,013	-0,043	-0,034	0,044	-0,096	-0,061
35. Satisfaction with health	0,270	0,280	-0,064	-0,070	0,084	0,048	-0,113	0,060	0,228
36. Disability	0,031	0,062	-0,027	-0,015	0,074	-0,003	-0,017	0,032	0,027

10	11	12	13	14	15	16	17	18	19	20	21	22
-0,076												
-0,095	-0,089											
0,623	-0,049	-0,060										
0,638	-0,048	-0,061	0,012									
-0,111	0,114	0,036	-0,070	-0,071								
-0,062	0,074	0,023	-0,039	-0,039	-0,096							
-0,037	0,062	0,028	-0,024	-0,023	-0,053	-0,026						
0,495	-0,037	-0,047	0,306	0,288	-0,055	-0,031	-0,018					
0,323	-0,024	-0,031	0,199	0,190	-0,036	-0,020	-0,012	0,022				
0,275	-0,021	-0,027	0,172	0,146	-0,031	-0,017	-0,010	0,019	0,013			
-0,007	-0,139	0,049	-0,003	-0,004	-0,176	-0,052	-0,024	-0,003	-0,002	-0,002		
0,000	-0,170	0,111	0,001	0,000	-0,021	0,005	0,006	0,000	0,000	0,000	0,616	
-0,002	0,002	0,001	-0,208	0,230	0,001	0,001	0,001	-0,048	-0,019	0,011	-0,001	-0,001
0,002	-0,059	-0,023	-0,012	0,005	0,153	0,186	0,166	0,017	0,037	0,061	0,479	0,530
0,007	0,059	0,097	0,005	0,015	-0,083	-0,101	-0,063	-0,010	-0,017	-0,014	-0,156	-0,071
0,008	0,000	-0,036	0,010	-0,002	0,082	0,087	0,060	0,009	0,008	0,001	0,103	0,077
-0,087	0,020	0,013	-0,054	-0,055	0,058	0,079	0,030	-0,043	-0,028	-0,024	-0,025	-0,007
-0,086	0,133	0,111	-0,056	-0,053	0,104	0,104	0,089	-0,042	-0,028	-0,024	-0,128	-0,066
-0,079	0,218	0,110	-0,049	-0,051	0,040	0,032	0,043	-0,039	-0,026	-0,022	-0,074	-0,090
-0,076	-0,018	-0,009	-0,060	-0,049	0,024	0,011	0,012	-0,035	-0,020	-0,016	0,036	-0,001
-0,074	-0,014	-0,032	-0,046	-0,047	0,007	0,017	0,012	-0,037	-0,024	-0,020	0,020	-0,001
-0,357	0,031	0,046	-0,263	-0,236	0,114	0,096	0,071	-0,163	-0,095	-0,073	0,145	0,120
-0,111	0,010	-0,048	-0,107	-0,066	0,138	0,202	0,229	-0,029	0,019	0,046	-0,049	-0,046
-0,070	0,029	-0,036	-0,006	-0,077	0,070	0,138	0,212	0,011	0,098	0,215	-0,102	-0,053
-0,010	0,023	-0,012	0,001	-0,011	-0,035	-0,028	-0,032	-0,010	-0,012	-0,017	-0,092	-0,115
-0,019	0,015	-0,009	-0,004	-0,013	-0,003	-0,010	-0,022	-0,008	-0,011	-0,018	-0,012	-0,052

23	24	25	26	27	28	29	30	31	32	33	34	35
0,020												
-0,006	-0,200											
-0,003	0,222	-0,338										
0,000	0,015	-0,032	0,005									
0,002	0,031	0,112	-0,115	-0,126								
-0,001	-0,050	0,080	-0,053	0,003	-0,038							
0,001	0,013	-0,091	0,079	-0,019	-0,063	-0,005						
0,000	0,044	-0,043	0,026	-0,013	-0,006	-0,085	-0,001					
0,004	0,152	-0,299	0,295	0,029	-0,035	-0,091	0,156	0,097				
0,012	0,450	-0,226	0,151	0,021	0,040	-0,003	0,035	0,048	0,161			
-0,178	0,331	-0,082	0,050	0,015	0,090	0,019	0,006	0,022	0,058	0,528		
-0,005	-0,200	0,036	-0,032	-0,003	-0,005	0,010	0,011	-0,006	-0,012	-0,089	-0,071	
-0,006	-0,091	-0,043	-0,002	0,006	-0,034	0,037	0,016	0,018	0,012	-0,041	-0,042	0,050
