# Supplementary material for: Do workers accumulate resources during continuous employment and lose them during unemployment, and what does that mean for their subjective well-being?
Source: PLoS One. 2021 Dec 23;16(12):e0261794. doi: 10.1371/journal.pone.0261794 (PMC8699683; doi:10.1371/journal.pone.0261794)
Supplement: S2 Table — (PDF) [file pone.0261794.s002.pdf]

Predictors	Income (logged)	Financial worries	Perceived employability	Frequency of socializing	Loneliness	Social support availability	Mastery
Within							
Unemployment occasion[a]	-0.270 **	0.742 **	-0.577 **	0.158	0.341 **	-0.243	-0.806 **
	[-0.296, -0.245]	[0.665, 0.819]	[-0.656, -0.499]	[-0.218, 0.534]	[0.203, 0.492]	[-0.573, 0.045]	[-1.071, -0.541]
First 6 months in current job	-0.014 **	0.073 **	0.053 **	0.182 **	-0.034	-0.008	0.034
	[-0.021, -0.006]	[0.050, 0.097]	[0.026, 0.080]	[0.077, 0.288]	[-0.085, 0.020]	[-0.133, 0.120]	[-0.038, 0.106]
Last year in current job	-0.030 **	0.133 **	0.087 **	0.181 **	-0.024	0.065	-0.098 **
	[-0.036, -0.025]	[0.113, 0.153]	[0.063, 0.111]	[0.092, 0.271]	[-0.073, 0.021]	[-0.034, 0.153]	[-0.159, -0.037]
First 6 months of current unemployment	0.033 *	-0.176 **	0.272 **	0.049	-0.170 *	0.237	0.306 *
	[0.007, 0.059]	[-0.257, -0.096]	[0.192, 0.351]	[-0.331, 0.429]	[-0.334, -0.014]	[-0.077, 0.615]	[0.062, 0.550]
Last year of current unemployment	0.029 *	-0.186 **	0.076	0.187	-0.169	0.135	0.186
	[0.004, 0.054]	[-0.273, -0.101]	[-0.009, 0.160]	[-0.220, 0.594]	[-0.345, 0.008]	[-0.186, 0.468]	[-0.071, 0.444]
2nd employment spell[b]	-0.068 **	-0.066 **	-0.103 **	-0.238 **	0.015	0.004	0.126 *
	[-0.081, -0.054]	[-0.093, -0.040]	[-0.135, -0.071]	[-0.404, -0.072]	[-0.044, 0.064]	[-0.099, 0.105]	[0.017, 0.235]
3rd employment spell[b]	-0.111 **	-0.052 *	-0.140 **	-0.295 *	0.025	0.003	0.181 *
	[-0.131, -0.091]	[-0.093, -0.010]	[-0.189, -0.091]	[-0.539, -0.051]	[-0.068, 0.103]	[-0.166, 0.142]	[0.017, 0.344]
4th and later employment spells[b]	-0.139 **	-0.109 **	-0.163 **	-0.345	0.030	0.165	0.189
	[-0.169, -0.109]	[-0.171, -0.048]	[-0.235, -0.090]	[-0.709, 0.020]	[-0.115, 0.173]	[-0.129, 0.418]	[-0.052, 0.430]
2nd unemployment spell[c]	-0.025 *	0.065	-0.011	0.166	-0.021	-0.202	-0.070
	[-0.049, -0.001]	[-0.004, 0.131]	[-0.078, 0.058]	[-0.143, 0.475]	[-0.151, 0.112]	[-0.481, 0.078]	[-0.269, 0.130]
3rd unemployment spell[c]	-0.035 *	0.124 *	0.058	0.113	-0.028	-0.080	0.090
	[-0.067, -0.004]	[0.021, 0.227]	[-0.040, 0.156]	[-0.330, 0.556]	[-0.235, 0.186]	[-0.437, 0.335]	[-0.200, 0.381]
4th and later unemployment spells[c]	-0.054	-0.047	-0.052	0.366	-0.231	0.339	-0.019
	[-0.114, 0.007]	[-0.175, 0.080]	[-0.179, 0.073]	[-0.257, 0.989]	[-0.529, 0.088]	[-0.138, 0.882]	[-0.424, 0.386]
**Employment duration**	0.000	-0.004 **	-0.009 **	-0.008	0.002	0.004	0.000
	[-0.001, 0.001]	[-0.006, -0.002]	[-0.011, -0.007]	[-0.019, 0.002]	[-0.003, 0.006]	[-0.004, 0.013]	[-0.007, 0.007]
**Organizational tenure**	0.002 **	0.004 **	-0.017 **	0.011 *	0.001	-0.008	-0.008 *
	[0.001, 0.003]	[0.002, 0.006]	[-0.019, -0.015]	[0.001, 0.020]	[-0.004, 0.005]	[-0.020, 0.003]	[-0.015, -0.002]
**Unemployment duration**	-0.036 **	0.088 **	-0.040 *	-0.022	0.041	-0.131 *	-0.055
	[-0.046, -0.026]	[0.056, 0.120]	[-0.072, -0.009]	[-0.169, 0.125]	[-0.026, 0.103]	[-0.252, -0.010]	[-0.143, 0.033]
Age	0.027 **	-0.004 **	-0.044 **	-0.072 **	0.020 **	-0.056 **	-0.034 **
	[0.026, 0.028]	[-0.006, -0.002]	[-0.046, -0.041]	[-0.085, -0.059]	[0.015, 0.025]	[-0.067, -0.046]	[-0.043, -0.025]
$Age^2/10$	0.000 **	-0.006 **	-0.010 **	0.022 **	0.001	0.001	0.003 *
	[0.000, 0.001]	[-0.007, -0.006]	[-0.011, -0.009]	[0.018, 0.025]	[-0.001, 0.002]	[-0.002, 0.004]	[0.001, 0.005]
Educational attainment	0.011 **	-0.009 *	0.059 **	0.053 *	-0.003	0.022	0.018
	[0.006, 0.015]	[-0.018, -0.001]	[0.049, 0.070]	[0.008, 0.098]	[-0.025, 0.018]	[-0.030, 0.073]	[-0.009, 0.045]
Part-time employment[d]	-0.081 **	0.052 **	-0.015	0.115 *	0.035	0.080	-0.127 **

	[-0.088, -0.073]	[0.029, 0.076]	[-0.043, 0.013]	[0.004, 0.226]	[-0.033, 0.095]	[-0.022, 0.195]	[-0.203, -0.052]
Marginal employment[d]	-0.134 **	0.214 **	-0.045 **	0.374 **	0.083 *	0.019	-0.219 **
	[-0.143, -0.125]	[0.189, 0.240]	[-0.076, -0.015]	[0.249, 0.498]	[0.020, 0.144]	[-0.144, 0.138]	[-0.301, -0.138]
Fixed-term contract	-0.030 **	0.119 **	-0.103 **	0.018	0.054 *	-0.029	-0.085 *
	[-0.037, -0.023]	[0.096, 0.142]	[-0.129, -0.078]	[-0.086, 0.122]	[0.003, 0.116]	[-0.198, 0.080]	[-0.158, -0.012]
Overtime hours	0.003 **	-0.005 **	0.007 **	-0.021 **	0.004	-0.001	0.006
	[0.003, 0.004]	[-0.007, -0.003]	[0.005, 0.010]	[-0.030, -0.011]	[-0.001, 0.009]	[-0.012, 0.010]	[0.000, 0.013]
Self-employment	-0.008	0.172 **	-0.085 **	-0.339 **	-0.043	-0.103	0.089
	[-0.023, 0.008]	[0.139, 0.205]	[-0.124, -0.045]	[-0.512, -0.165]	[-0.116, 0.037]	[-0.247, 0.046]	[-0.026, 0.204]
Occupational autonomy	0.035 **	-0.058 **	-0.015 **	0.103 **	-0.023 *	-0.002	0.048 **
	[0.031, 0.038]	[-0.067, -0.049]	[-0.026, -0.005]	[0.059, 0.148]	[-0.045, -0.004]	[-0.049, 0.042]	[0.019, 0.078]
Number of prior unemployment spells	0.002	0.040 **	0.022	0.002	0.006	-0.085	0.024
	[-0.007, 0.010]	[0.020, 0.061]	[-0.001, 0.045]	[-0.106, 0.111]	[-0.040, 0.057]	[-0.189, 0.035]	[-0.049, 0.097]
Total duration of prior unemployment	-0.026 **	0.023 **	0.018 **	0.001	0.018	-0.021	-0.035
	[-0.031, -0.021]	[0.012, 0.035]	[0.006, 0.029]	[-0.066, 0.069]	[-0.009, 0.044]	[-0.071, 0.026]	[-0.077, 0.008]
Satisfaction with health	0.003 **	-0.050 **	0.026 **	0.089 **	-0.066 **	0.007	0.140 **
	[0.002, 0.004]	[-0.054, -0.047]	[0.022, 0.030]	[0.072, 0.106]	[-0.075, -0.057]	[-0.012, 0.023]	[0.124, 0.155]
Disability	0.006	-0.036 *	0.194 **	-0.101	0.000[f]	0.000 [f]	-0.072
	[-0.004, 0.016]	[-0.071, -0.005]	[0.156, 0.234]	[-0.270, 0.068]			[-0.171, 0.027]
Residual variance (σ^2_e)	0.068 **	N/A	N/A	17.102 **	N/A	N/A	1.000[f]
	[0.066, 0.071]			[16.902, 17.303]			
R^2	.256	N/A	N/A	.027	N/A	N/A	.128
Between							
Unemployed at all occasions	-0.130 **	0.007	0.054	0.005	-0.084	0.012	-0.356
	[-0.183, -0.077]	[-0.153, 0.165]	[-0.117, 0.222]	[-0.756, 0.765]	[-0.565, 0.399]	[-0.649, 0.613]	[-1.075, 0.364]
Number of employment spells[e]	-0.036 **	0.034 **	0.069 **	-0.030	0.006	0.012	-0.005
	[-0.042, -0.029]	[0.017, 0.051]	[0.049, 0.088]	[-0.090, 0.030]	[-0.018, 0.033]	[-0.037, 0.059]	[-0.042, 0.032]
Number of unemployment spells[e]	-0.044 **	0.036 **	-0.057 **	-0.176 **	-0.020	0.014	-0.077 **
	[-0.051, -0.037]	[0.016, 0.057]	[-0.081,-0.033]	[-0.254, -0.097]	[-0.049, 0.006]	[-0.037, 0.062]	[-0.120, -0.035]
Average employment duration	0.001 **	0.001	-0.002	0.007 *	0.000	0.000	0.002
	[0.000, 0.002]	[-0.001, 0.002]	[-0.004, 0.000]	[0.001, 0.014]	[-0.003, 0.002]	[-0.005, 0.004]	[-0.002, 0.007]
Average organizational tenure	0.001 *	-0.004 **	-0.026 **	0.014 **	-0.005 **	0.004	-0.005
	[0.000, 0.002]	[-0.006, -0.002]	[-0.028, -0.024]	[0.006, 0.022]	[-0.009, -0.002]	[-0.002, 0.009]	[-0.010, 0.000]
Average unemployment duration	-0.034 **	0.018	-0.088 **	-0.107 *	-0.005	-0.015	-0.074 *
	[-0.043, -0.026]	[-0.007, 0.043]	[-0.116, -0.059]	[-0.209, -0.005]	[-0.049, 0.037]	[-0.076, 0.051]	[-0.137, -0.010]
Age	0.002 **	-0.007 **	-0.042 **	-0.113 **	-0.005 **	0.002	-0.024 **
	[0.001, 0.002]	[-0.009, -0.006]	[-0.044, -0.040]	[-0.118, -0.107]	[-0.007, -0.003]	[-0.002, 0.006]	[-0.029, -0.020]
Age^2/10	0.001 **	-0.007 **	-0.011 **	0.027 **	0.002 *	0.001	0.006 **

	[0.001, 0.002]	[-0.008, -0.006]	[-0.012, -0.010]	[0.024, 0.031]	[0.000, 0.003]	[-0.002, 0.003]	[0.004, 0.008]
East German	-0.106 **	0.447 **	-0.354 **	-0.952 **	-0.016	0.114 **	-0.082 *
	[-0.118, -0.094]	[0.422, 0.473]	[-0.384, -0.323]	[-1.074, -0.831]	[-0.051, 0.020]	[0.037, 0.179]	[-0.157, -0.008]
Woman	0.047 **	0.046 **	-0.062 **	0.066	0.133 **	0.082 **	-0.011
	[0.038, 0.056]	[0.023, 0.070]	[-0.091, -0.035]	[-0.018, 0.150]	[0.099, 0.166]	[0.018, 0.155]	[-0.065, 0.044]
Average educational attainment	0.034 **	-0.050 **	0.022 **	0.084 **	0.011 *	0.002	0.030 **
	[0.031, 0.037]	[-0.055, -0.044]	[0.015, 0.029]	[0.059, 0.110]	[0.003, 0.019]	[-0.012, 0.016]	[0.012, 0.047]
Rate of part-time employment	0.013	-0.146 **	0.235 **	1.085 **	-0.062	-0.044	0.123
	[-0.015, 0.041]	[-0.202, -0.091]	[0.168, 0.301]	[0.823, 1.348]	[-0.170, 0.051]	[-0.211, 0.134]	[-0.021, 0.268]
Rate of marginal employment	-0.048 **	-0.259 **	0.321 **	1.098 **	0.100	0.002	0.302 **
	[-0.078, -0.017]	[-0.315, -0.204]	[0.254, 0.390]	[0.831, 1.364]	[-0.021, 0.206]	[-0.180, 0.165]	[0.133, 0.471]
Rate of fixed-term employment	0.216 **	0.008	0.049	0.371	0.099	-0.215	0.225
	[0.173, 0.259]	[-0.053, 0.068]	[-0.023, 0.121]	[-0.023, 0.765]	[-0.033, 0.204]	[-0.399, 0.019]	[-0.016, 0.466]
Average overtime hours	0.013 **	0.012 **	0.036 **	-0.030	0.003	-0.013 *	0.033 **
	[0.009, 0.017]	[0.007, 0.017]	[0.030, 0.042]	[-0.061, 0.001]	[-0.005, 0.012]	[-0.027, -0.001]	[0.015, 0.051]
Rate of self-employment	-0.061 **	0.210 **	0.151 **	-0.514 **	-0.016	-0.047	0.227 **
	[-0.091, -0.030]	[0.152, 0.268]	[0.078, 0.222]	[-0.735, -0.293]	[-0.120, 0.084]	[-0.263, 0.133]	[0.094, 0.360]
Average occupational autonomy	0.126 **	-0.162 **	0.018	0.248 **	-0.055 **	0.053 *	0.294 **
	[0.117, 0.135]	[-0.178, -0.147]	[0.000, 0.037]	[0.167, 0.329]	[-0.079, -0.032]	[0.013, 0.093]	[0.235, 0.353]
Average number of prior	-0.013 **	0.049 **	-0.049 **	-0.032	0.037 *	-0.030	-0.011
unemployment spells	[-0.021, -0.005]	[0.027, 0.070]	[-0.074, -0.024]	[-0.122, 0.057]	[0.006, 0.070]	[-0.086, 0.021]	[-0.062, 0.039]
Average duration of prior	-0.018 **	0.040 **	-0.030 **	-0.008	0.016	-0.025 *	-0.035 *
unemployment	[-0.022, -0.014]	[0.029, 0.051]	[-0.042, -0.017]	[-0.057, 0.042]	[-0.003, 0.033]	[-0.050, -0.003]	[-0.063, -0.006]
Average satisfaction with health	0.024 **	-0.146 **	0.081 **	0.267 **	-0.199 **	0.063 **	0.436 **
	[0.020, 0.029]	[-0.154, -0.139]	[0.072, 0.089]	[0.225, 0.308]	[-0.212, -0.187]	[0.045, 0.083]	[0.396, 0.476]
Disability at all occasions	-0.033 *	-0.161 **	-0.237 **	-0.286	-0.004	0.119	0.131
	[-0.061, -0.006]	[-0.236, -0.086]	[-0.329, -0.147]	[-0.614, 0.043]	[-0.143, 0.111]	[-0.065, 0.348]	[-0.081, 0.343]
Intercept/Threshold 1	6.281 **	-0.795 **	-1.136 **	6.402 **	0.258 **	-2.286 **	0.000[f]
	[6.235, 6.327]	[-0.880, -0.718]	[-1.204, -1.053]	[5.889, 6.916]	[0.189, 0.336]	[-2.437, -2.161]	
Threshold 2	N/A	1.181 **	1.369 **	N/A	1.531 **	N/A	N/A
		[1.096, 1.257]	[1.301, 1.454]		[1.459, 1.609]		
Threshold 3	N/A	N/A	N/A	N/A	2.243 **	N/A	N/A
					[2.167, 2.321]		
Residual variance (σ^2_u)	0.109 **	0.636 **	0.873 **	7.444 **	0.479 **	0.647 **	1.000[f]
	[0.103, 0.114]	[0.619, 0.653]	[0.850, 0.896]	[7.224, 7.664]	[0.454, 0.508]	[0.547, 0.817]	
R^2	.335	N/A	N/A	0.337	N/A	N/A	.432

Note. For all analyses, N_{persons} = 45,526, $N_{\text{observations}}$ = 317,236. Cells represent unstandardized regression coefficients with 95% confidence intervals (credibility intervals for Bayesian estimates) in square brackets. For income, frequency of socializing, and mastery, linear regression analyses with MLR estimation were conducted. For all other DVs, probit regression analyses with Bayeasian estimation were conducted.

[a] Vs. employment occasion
[b] Vs. 1st employment spell (during the observation period)
[c] Vs. 1st unemployment spell (during the observation period)
[d] Vs. full-time employment
[e] During the observation period (in contrast, prior unemployment spells could also include those prior to the observation period)
[f] Residual variance of the latent indicator of mastery was fixed at 1.0 at both levels, whereas factor loadings were freely estimated
* $p < .05$. ** $p < .01$.
