# Supplementary material for: Do workers accumulate resources during continuous employment and lose them during unemployment, and what does that mean for their subjective well-being?
Source: PLoS One. 2021 Dec 23;16(12):e0261794. doi: 10.1371/journal.pone.0261794 (PMC8699683; doi:10.1371/journal.pone.0261794)
Supplement: S3 Table — (PDF) [file pone.0261794.s003.pdf]

Effects	Life satisfaction *B*	95 % CI	Emotional well-being *B*	95 % CI
Within				
Unemployment occasion[a]	-0.538 **	[-0.647, -0.429]	-0.024	[-0.280, 0.231]
First 6 months in current job	-0.072 **	[-0.100, -0.045]	-0.096 *	[-0.170, -0.023]
Last year in current job	-0.105 **	[-0.128, -0.083]	-0.108 **	[-0.174, -0.042]
First 6 months of current unemployment	-0.024	[-0.130, 0.082]	-0.277 *	[-0.517, -0.037]
Last year of current unemployment	0.032	[-0.076, 0.140]	-0.220	[-0.473, 0.034]
2nd employment spell[b]	-0.014	[-0.056, 0.029]	0.044	[-0.070, 0.158]
3rd employment spell[b]	0.017	[-0.048, 0.082]	0.105	[-0.061, 0.270]
4th and later employment spells[b]	-0.036	[-0.134, 0.063]	0.112	[-0.122, 0.345]
2nd unemployment spell[c]	0.042	[-0.048, 0.133]	-0.058	[-0.274, 0.158]
3rd unemployment spell[c]	0.065	[-0.068, 0.198]	-0.003	[-0.297, 0.291]
4th and later unemployment spells[c]	0.191 *	[0.013, 0.368]	0.145	[-0.169, 0.459]
**Employment duration**	0.003 *	[0.001, 0.006]	0.004	[-0.003, 0.011]
**Organizational tenure**	-0.001	[-0.003, 0.002]	-0.002	[-0.010, 0.005]
**Unemployment duration**	0.019	[-0.020, 0.058]	0.050	[-0.037, 0.137]
**Income**	0.229 **	[0.200, 0.258]	-0.002	[-0.074, 0.070]
**Financial worries**	-0.076 **	[-0.095, -0.056]	-0.116 **	[-0.164, -0.069]
**Perceived employability**	-0.012	[-0.028, 0.003]	-0.065 **	[-0.102, -0.028]
**Frequency of socializing**	0.006 **	[0.004, 0.008]	0.010 **	[0.005, 0.015]
**Social support availability**	0.382 **	[0.299, 0.465]	0.322 **	[0.146, 0.498]
**Mastery**	0.486 **	[0.458, 0.513]	0.522 **	[0.438, 0.606]
Age	0.003	[-0.001, 0.007]	0.015 **	[0.004, 0.025]
$Age^2/10$	0.003 **	[0.002, 0.004]	0.001	[-0.003, 0.004]
Educational attainment	-0.020 **	[-0.033, -0.007]	0.004	[-0.040, 0.048]
Part-time employment[d]	-0.039	[-0.080, 0.001]	0.010	[-0.067, 0.087]
Marginal employment[d]	-0.136 **	[-0.181, -0.090]	-0.065	[-0.151, 0.021]
Fixed-term contract	-0.018	[-0.057, 0.020]	-0.042	[-0.115, 0.031]
Overtime hours	-0.004 *	[-0.008, -0.001]	-0.010 **	[-0.017, -0.004]
Self-employment	-0.111 **	[-0.169, -0.053]	-0.037	[-0.160, 0.086]
Occupational autonomy	0.009	[-0.007, 0.025]	-0.007	[-0.041, 0.027]
Number of prior unemployment spells	0.024	[-0.015, 0.062]	-0.056	[-0.138, 0.026]
Total duration of prior unemployment	0.034 **	[0.011, 0.058]	0.030	[-0.014, 0.074]
Satisfaction with health	0.169 **	[0.161, 0.177]	0.153 **	[0.139, 0.167]
Disability	0.049	[-0.005, 0.104]	0.236 **	[0.131, 0.341]
Residual variance (σ^2_e)	1.282 **	[1.252, 1.311]	1.000[f]	
R^2	.250		.293	
Between				
Unemployed at all occasions	-0.063	[-0.296, 0.169]	0.120	[-0.267, 0.508]
Number of employment spells[e]	-0.039 **	[-0.056, -0.022]	-0.049	[-0.100, 0.002]
Number of unemployment spells[e]	-0.037 **	[-0.057, -0.016]	0.023	[-0.023, 0.069]
Average employment duration	0.001	[0.000, 0.003]	-0.001	[-0.005, 0.003]
Average organizational tenure	0.004 **	[0.002, 0.006]	-0.007 **	[-0.012, -0.003]
Average unemployment duration	0.039 **	[0.010, 0.068]	0.048	[-0.010, 0.106]
Income	0.196 **	[0.150, 0.242]	-0.093	[-0.197, 0.011]
Financial worries	-0.186 **	[-0.248, -0.124]	-0.377 **	[-0.498, -0.257]
Perceived employability	-0.173 **	[-0.217, -0.129]	-0.269 **	[-0.361, -0.178]
Frequency of socializing	0.013 **	[0.008, 0.019]	-0.010	[-0.023, 0.002]
Social support availability	1.941 **	[1.458, 2.425]	2.599 **	[1.744, 3.455]
Mastery	0.571 **	[0.531, 0.612]	0.551 **	[0.451, 0.650]
Age	0.018 **	[0.016, 0.020]	0.027 **	[0.023, 0.032]
$Age^2/10$	-0.001	[-0.002, 0.000]	-0.003 *	[-0.005, 0.000]
East German	-0.285 **	[-0.323, -0.246]	0.119 **	[0.050, 0.188]
Woman	0.041 **	[0.013, 0.069]	-0.822 **	[-0.884, -0.760]
Average educational attainment	-0.024 **	[-0.031, -0.016]	-0.029 **	[-0.043, -0.014]

Rate of part-time employment	0.156 **	[0.079, 0.234]	0.020	[-0.130, 0.171]
Rate of marginal employment	0.112 *	[0.026, 0.198]	-0.430 **	[-0.589, -0.270]
Rate of fixed-term employment	-0.010	[-0.136, 0.115]	-0.252 *	[-0.474, -0.030]
Average overtime hours	-0.022 **	[-0.031, -0.013]	-0.017	[-0.035, 0.000]
Rate of self-employment	-0.244 **	[-0.315, -0.172]	-0.117	[-0.244, 0.010]
Average occupational autonomy	-0.035 **	[-0.060, -0.009]	-0.111 **	[-0.159, -0.062]
Average number of prior unemployment spells	-0.023	[-0.050, 0.004]	-0.002	[-0.053, 0.049]
Average duration of prior unemployment	-0.012	[-0.028, 0.004]	0.024	[-0.002, 0.050]
Average satisfaction with health	0.333 **	[0.316, 0.350]	0.353 **	[0.317, 0.388]
Disability at all occasions	0.128 *	[0.022, 0.235]	0.244 *	[0.039, 0.449]
Intercept	4.401 **	[4.004, 4.797]	0.000[f]	
Residual variance (σ^2_u)	0.393 **	[0.372, 0.413]	1.000[f]	
R^2	.697		.501	

Note. For all analyses, N_{persons} = 45,526, $N_{\text{observations}}$ = 317,236. Number of (un)employment spells and average durations of employment, unemployment, and organizational tenure were grand-mean centered. Age was centered at 43 years of age.

[a] Vs. employment occasion

[b] Vs. 1st employment spell (during the period of observations)

[c] Vs. 1st unemployment spell (during the period of observations)

[d] Vs. full-time employment

[e] During the observation period (in contrast, prior unemployment spells could also include those prior to the observation period)

[f] Residual variance of the latent indicator of emotional well-being was fixed at 1.0 at both levels, whereas factor loadings were freely estimated

* $p < .05$. ** $p < .01$.
