# Supplementary material for: Do workers accumulate resources during continuous employment and lose them during unemployment, and what does that mean for their subjective well-being?
Source: PLoS One. 2021 Dec 23;16(12):e0261794. doi: 10.1371/journal.pone.0261794 (PMC8699683; doi:10.1371/journal.pone.0261794)
Supplement: Attachment — Submitted filename: Review Comment for PLOUS one.docx [file pone.0261794.s004.docx]

Review Comment

The manuscript is judged to be meaningful as data analyzed using objective indicators in various fields. However, it is hoped that the manuscript will be improved through corrections in some parts.

1) Overall, it provides a lot of information and is considered to be useful, but I hope that it will be condensed.
2) In relation to well-being, age, gender, and SES were considered, but other variables were not considered. Why? For example, if an industrial accident occurs, the results of this study may differ, and this needs to be considered. Although such a study may be meaningful, it is judged that the results may be meaningless if the missing parameters are considered.
3) It would be good if you divide the manuscript into introduction, theory, method, result and discussion, and conclusions. In particular, there is a disadvantage that the introduction part is too long and the research result part looks weak.
