# Supplementary material for: Do workers accumulate resources during continuous employment and lose them during unemployment, and what does that mean for their subjective well-being?
Source: PLoS One. 2021 Dec 23;16(12):e0261794. doi: 10.1371/journal.pone.0261794 (PMC8699683; doi:10.1371/journal.pone.0261794)
Supplement: Attachment — Submitted filename: Response to reviewers.docx [file pone.0261794.s005.docx]

**Response to the decision letter**

**Journal Requirements:**

1. When submitting your revision, we need you to address these additional requirements.

Please ensure that your manuscript meets PLOS ONE's style requirements, including those for file naming. The PLOS ONE style templates can be found at
<https://journals.plos.org/plosone/s/file?id=wjVg/PLOSOne_formatting_sample_main_body.pdf> and
<https://journals.plos.org/plosone/s/file?id=ba62/PLOSOne_formatting_sample_title_authors_affiliations.pdf>

*I have completely revised the manuscript to be in line with PLOS ONE style. Among other things, I converted footnotes into main text, which shows in the markup.*

2. In your Data Availability statement, you have not specified where the minimal data set underlying the results described in your manuscript can be found. PLOS defines a study's minimal data set as the underlying data used to reach the conclusions drawn in the manuscript and any additional data required to replicate the reported study findings in their entirety. All PLOS journals require that the minimal data set be made fully available. For more information about our data policy, please see <http://journals.plos.org/plosone/s/data-availability>.

Upon re-submitting your revised manuscript, please upload your study’s minimal underlying data set as either Supporting Information files or to a stable, public repository and include the relevant URLs, DOIs, or accession numbers within your revised cover letter. For a list of acceptable repositories, please see <http://journals.plos.org/plosone/s/data-availability#loc-recommended-repositories>. Any potentially identifying patient information must be fully anonymized.

Important: If there are ethical or legal restrictions to sharing your data publicly, please explain these restrictions in detail. Please see our guidelines for more information on what we consider unacceptable restrictions to publicly sharing data: <http://journals.plos.org/plosone/s/data-availability#loc-unacceptable-data-access-restrictions>. Note that it is not acceptable for the authors to be the sole named individuals responsible for ensuring data access.

We will update your Data Availability statement to reflect the information you provide in your cover letter.

*In the original submission, I specified:*

*“The data underlying the results presented in the study are available from the DIW Berlin via a data distribution contract. The dataset doi: https://doi.org/10.5684/soep.v30.”*

*The SOEP data are owned by a third party (DIW Berlin) but are freely accessible to all researchers who register themselves as users. I may not share the data, not even my working dataset. Please let me know whether I should modify my Data Availability statement or do something else.*

3. Please include captions for your Supporting Information files at the end of your manuscript, and update any in-text citations to match accordingly. Please see our Supporting Information guidelines for more information: <http://journals.plos.org/plosone/s/supporting-information>.

*Done.*

4. Please review your reference list to ensure that it is complete and correct. If you have cited papers that have been retracted, please include the rationale for doing so in the manuscript text, or remove these references and replace them with relevant current references. Any changes to the reference list should be mentioned in the rebuttal letter that accompanies your revised manuscript. If you need to cite a retracted article, indicate the article’s retracted status in the References list and also include a citation and full reference for the retraction notice.

*I have checked all references, there are no retracted or corrected articles in the list.*

**Reviewers' comments:**

Reviewer #1: 1. The revised introduction should motivate the empirical context (i.e., focus on Germany).

*In the revision, I moved the paragraph on the German context from “The Present Study” to the Introduction and added a sentence that explains why this context is interesting for my research questions (pp. 3-4).*

2. Is attrition an issue in the data? Individuals with lowest level of health status may be much more likely to drop from the panel over time. Does this have implications for the interpretation of the results that are presented in the paper?

*I used satisfaction with health from the previous measurement as one of missing value covariates (see p. 23). In addition, wherever possible (i.e., with maximum likelihood estimation but not with Bayesian estimation), I included the dependent variable from the previous wave as a missing value covariate. As most dependent variables referred to well-being, they can be seen as mental health indicators. In the revision, I added to Limitations (pp. 44-45):*

*“Even though I applied full information estimators and included missing data covariates, attrition might influence the present findings. For example, the effects of long-term unemployment might be underestimated if those unemployed individuals who experienced substantial mental health deterioration systematically dropped out of the panel.”*

3. Is educational attainment predetermined control variable or not?

*Educational attainment was always a control variable at both within and between levels, even in the models where average educational attainment served as a moderator at the between level. To make it clearer, I added in the revision (see p. 22):*

*“All time-varying control variables were assessed yearly. Most of them (except for the characteristics of the current spell, which were purely within-level variables) were also included as control variables at the between level (see S2 Table for a full list of control variables at both levels).”*

4. Is there any need to account for local economic conditions that may be relevant for both health and employment status?

*It is possible that there is regional variation in the effects. However, addressing it would go beyond the scope of the present study, which focused on the individual level (and is even so very extensive). In the revision, I added to future directions (p. 46):*

*“At the macro level, the consequences of long-term employment or unemployment may be compared across different regional and country contexts… Even within one country, varying regional economic conditions may lead to unemployment being more or less normative and employment being more or less rewarding.”*

5. There is earlier empirical literature on health and labor market status in health economics using panel data (<https://doi.org/10.1002/hec.1361>). This issue should be acknowledged in the revised version.

*Thank you for sharing this reference. It is highly relevant indeed, and I have cited it multiple times in the Introduction and Discussion.*

6. What are the key policy lessons for other countries? What is the external validity of the estimates?

*I added to Conclusions (p. 47): “In general, the highly regulated German labor market and social security system may both dampen the rewards of a strong labor force attachment and buffer against the losses of long-term unemployment. Still, my findings may be generalizable to other Western European countries with comparable labor market regimes (e.g., Austria, Belgium, or the Netherlands). A policy recommendation for such countries may be to loosen their employment regulations in a direction that would lead to more employment (and job change) opportunities to workers of different age and would better reward continuous employment.”*

Reviewer #2: The manuscript is judged to be meaningful as data analyzed using objective indicators in various fields. However, it is hoped that the manuscript will be improved through corrections in some parts.

1) Overall, it provides a lot of information and is considered to be useful, but I hope that it will be condensed.

*As suggested by the Academic Editor, I did not condense the manuscript. I should note that I did shorten the manuscript previously, but it remains so long because of the complexity of the research questions and data analyses and also because I wanted to avoid piecemeal publication of findings.*

2) In relation to well-being, age, gender, and SES were considered, but other variables were not considered. Why? For example, if an industrial accident occurs, the results of this study may differ, and this needs to be considered. Although such a study may be meaningful, it is judged that the results may be meaningless if the missing parameters are considered.

*This is very true, there may be multiple factors and events that might influence accumulation or depletion of resources and well-being. However, I considered an extensive set of control variables at both levels (see p. 22). Among other things, I controlled for health satisfaction and disability – both at the current measurement occasion at the within level and on average (or, in case of disability, on all occasions vs. only some) at the between level. I believe that the effects of an industrial accident would be reflected in a change in these variables, which are controlled for. As regards my choice of moderators (age, gender, SES), it was purely theoretically driven, because CAD theory attempts to explain inequality, whereas these variables are major factors of inequality.*

3) It would be good if you divide the manuscript into introduction, theory, method, result and discussion, and conclusions. In particular, there is a disadvantage that the introduction part is too long and the research result part looks weak.

*I corrected the revision to be in line with PLOS One style guidelines. The apparent weakness of the research part might have to do with tables and figures being absent from the manuscript body (in the original submission). In the revision, in line with PLOS style, tables are directly in the manuscript body and figure captions as well.*

**Additional changes**

*1. I spotted another useful reference and added it to the manuscript:*

*Stiglbauer B, Batinic B. The role of Jahoda’s latent and financial benefits for work involvement: A longitudinal study. J Vocat Behav. 2012;81: 259–268. doi:10.1016/j.jvb.2012.07.008*

*2. I changed figures from grayscale to color, because this is an online-only publication, and color makes figures better readable.*
